# Complete Genome Sequence of *Salmonella enterica* subsp. *enterica* Serovar Agona 460004 2-1, Associated with a Multistate Outbreak in the United States

**DOI:** 10.1128/genomeA.00690-15

**Published:** 2015-07-02

**Authors:** Maria Hoffmann, Justin Payne, Richard J. Roberts, Marc W. Allard, Eric W. Brown, James B. Pettengill

**Affiliations:** a Center for Food Safety and Nutrition, Division of Microbiology, Office of Regulatory Science, U.S. Food and Drug Administration, College Park, Maryland, USA; b New England Biolabs, Inc., Ipswich, Massachusetts, USA; c Center for Food Safety and Nutrition, Division of Public Health Informatics and Analytics, Office of Analytics and Outreach, U.S. Food and Drug Administration, College Park, Maryland, USA

## Abstract

Within the last several years, *Salmonella enterica* subsp. *enterica* serovar Agona has been among the 20 most frequently isolated serovars in clinical cases of salmonellosis. In this report, the complete genome sequence of *S*. Agona strain 460004 2-1 isolated from unsweetened puffed-rice cereal during a multistate outbreak in 2008 was sequenced using single-molecule real-time DNA sequencing.

## GENOME ANNOUNCEMENT

*Salmonella enterica* subsp. *enterica* serovar Agona was first isolated from cattle in Ghana in 1952 (1). Since then, it has been associated with several foodborne outbreaks worldwide. In April 2008, the Centers for Disease Control and Prevention (CDC) announced a 15-state outbreak of *S*. Agona infections resulting in 28 identified cases, with eight individuals being hospitalized. The federal investigation suggested that unsweetened puffed-rice cereals and unsweetened puffed-wheat cereals were the likely sources of contamination (2).

In this report, we announce the availability of a complete closed genome sequence of *S*. Agona 460004 2-1, which was isolated from unsweetened puffed-rice cereal in Minnesota (April 2008). The U.S. Food and Drug Administration obtained this isolate as part of a federal public health investigation during the multistate outbreak.

The *S*. Agona isolate was cultured in Trypticase soy broth (TSB) (Becton Dickinson, Franklin Lakes, NJ) overnight at 37°C. Genomic DNA was isolated from overnight cultures using the DNeasy blood and tissue kit (Qiagen, Inc., Valencia, CA). The pulsed-field gel electrophoresis (PFGE) showed that isolate 460004 2-1 has the Xbal pattern JABX01.0001 and the BlnI pattern JABA26.0001. The genome was sequenced using the Pacific Biosciences (PacBio) RS II sequencing platform, as previously reported (3, 4). A single SMRTbell 10-kb library was prepared according to the 10-kb PacBio sample preparation protocol and sequenced using C2 chemistry on two single-molecule real-time (SMRT) cells with a 180-min collection protocol. The 10-kb continuous long read (CLR) data were *de novo* assembled using the PacBio hierarchical genome assembly process (HGAP3)/Quiver software package, followed by Minimus2, and polished by Quiver (5). The assembled sequences were annotated using the NCBI Prokaryotic Genomes Annotation Pipeline (PGAP) and have been deposited at DDBJ/EMBL/GenBank.

Using the PacBio RS II system, we fully closed the genome, with a coverage of 134×. The genome size is 4,779,171 bp, with a G+C content of 52.1%, and it contains 4,636 genes. During sequencing, epigenetic modifications at each nucleotide position were measured as kinetic variations (KVs) in the nucleotide incorporation rates; methylase activities were deduced from the KV data (6). Six DNA methyltransferase recognition motifs were detected by SMRT sequencing, and the genes encoding the various motifs are shown in Table 1. The methylome of this isolate was analyzed and deposited in REBASE (http://tools.neb.com/~vincze/genomes/view.php?view_id=34334).

**TABLE 1:** Summary of methyltransferases identified in *S*. Agona 460004 2-1

Assignment	Methyltransferase specificity	Methylation type	Restriction modification type
M.SenA46Dcm	C**C**W**G**G	m5C	II
M.SenA46I	CAG**A**G	m6A	III
M.SenA46III	A**T**GC**A**T	m6A	II
M.SenA46IV	CA**GC**TG	m4C	II
M1.SenA46II	C**C**CNNNNNRTAG	m4C	I
M2.SenA46II	CCCNNNNNR**T**AG	m6A	I
M.SenA46DamP	GATC^a^	m6A	Orphan
M.SenA46ORF22330P	GATC^a^	m6A	II

a GATC cannot be matched unambiguously because there are 2 candidates. However, it is likely that M.SenA46DamP is active. M.SenA46ORF22330P is prophage encoded, which usually renders the gene inactive under normal growth conditions.

Further PHAST (7) analysis identified two intact prophages. One of the prophages had 72% overlap with 96% sequence similarity to prophage Fels-2, and the other intact prophage did not show any sequence similarity to any known phages when queried against the BLAST database. Both phages carry a type II methyltransferase.

### Nucleotide sequence accession number.

The complete genome sequence of *S*. Agona 460004 2-1 is available in GenBank under the accession no. CP011259.

## References

[1] Guinee PA, Kampelmacher EH, Willems HM 1961 Six new *Salmonella* types, isolated in Ghana (*S.* Volta, *S.* Agona, *S.* Wa, *S.* Techimani, *S.* Mampong and *S.* Tafo). Antonie van Leeuwenhoek 27:469–472. 13902866 10.1007/BF02538474

[2] CDC. 2008 Multistate outbreak of *Salmonella* Agona infections linked to rice & wheat puff cereal (final update). Centers for Disease Control and Prevention, Atlanta, GA http://www.cdc.gov/salmonella/agona/.

[3] Hoffmann M, Muruvanda T, Allard MW, Korlach J, Roberts RJ, Timme R, Payne J, McDermott PF, Evans P, Meng J, Brown EW, Zhao S 2013 Complete genome sequence of a multidrug-resistant *Salmonella enterica* serovar Typhimurium var. 5- strain isolated from chicken breast. Genome Announc 1(6):e01068-13. doi:10.1128/genomeA.01068-13. 24356834 PMC3868858

[4] Pirone-Davies C, Hoffmann M, Roberts RJ, Muruvanda T, Timme RE, Strain E, Luo Y, Payne J, Luong K, Song Y, Tsai YC, Boitano M, Clark TA, Korlach J, Evans PS, Allard MW 2015 Genome-wide methylation patterns in *Salmonella enterica* subsp. *enterica* serovars. PLoS One 10:e0123639. doi:10.1371/journal.pone.0123639. 25860355 PMC4393132

[5] Chin CS, Alexander DH, Marks P, Klammer AA, Drake J, Heiner C, Clum A, Copeland A, Huddleston J, Eichler EE, Turner SW, Korlach J 2013 Nonhybrid, finished microbial genome assemblies from long-read SMRT sequencing data. Nat Methods 10:563–569. doi:10.1038/nmeth.2474. 23644548

[6] Korlach J, Turner SW 2012 Going beyond five bases in DNA sequencing. Curr Opin Struct Biol 22:251–261. doi:10.1016/j.sbi.2012.04.002. 22575758

[7] Zhou Y, Liang Y, Lynch KH, Dennis JJ, Wishart DS 2011 PHAST: a fast phage search tool. Nucleic Acids Res 39:W347–W352. doi:10.1093/nar/gkr485. 21672955 PMC3125810

